# Rapid Dwarfing of an Insular Mammal – The Feral Cattle of Amsterdam Island

**DOI:** 10.1038/s41598-017-08820-2

**Published:** 2017-08-18

**Authors:** Roberto Rozzi, Mark V. Lomolino

**Affiliations:** 1 Museum für Naturkunde, Leibniz-Institut für Evolutions- und Biodiversitätsforschung, 10115 Berlin, Germany; 2 State University of New York, College of Environmental Science and Forestry, Syracuse, NY 13210 USA

## Abstract

The island rule describes a graded trend in insular populations of vertebrates from gigantism in small species to dwarfism in large species. The dwarfing of large mammals on islands has been observed both in the present fauna and in the fossil record. Elephants, hippopotami, deer, and other species became dwarfed on islands scattered all over the world, from the Mediterranean Sea to Indonesia, from the Eastern to Western Pacific Ocean, from the Caribbean to Canary Islands. The most rapid and well documented cases of island dwarfing known thus far took place over thousands of years. Here, we describe a rapid example of dwarfing of a large mammal - the feral cattle of Amsterdam Island, southern Indian Ocean, which dwarfed to about three quarters of its body size in slightly more than one century. This population provides us with a rare opportunity to assess the rapidity of demographic, life history, and morphological responses of large mammals to a very isolated and ecologically simple, insular environment.

## Introduction

The island rule describes a graded trend from gigantism in small species of vertebrates to dwarfism in large species^1–6^, and it is part of a more general suite of responses to the geographic isolation and ecological simplicity of islands known as the island syndrome^7^. In addition to shifts in body size towards that of intermediate-sized mammals, the island syndrome (*sensu lato*)^8^ includes demographic and ecological responses (insular populations exhibiting abnormally high densities and occurring in an unusually broad range of habitats), shifts in life history characteristics (e.g., altered generation times and reproductive potentials), and modifications of bauplans^9^ (e.g., towards that supporting low-gear locomotion in large mammals such as insular elephants and hippos).

These sometimes remarkable shifts in morphological, physiological, behavioral, and ecological characteristics of insular populations of mammals may ultimately be the products of the ecological simplicity of the islands they inhabit^3, 4^. Essentially, the island syndrome may well evidence reversals in natural selection from ecological displacement favouring diversification and adaptive radiations in species-rich communities, to ecological release in species-poor insular communities (in particular, those lacking mammalian competitors and predators) and convergence on the intermediate but absent phenotypes^2–4, 8^. Furthermore, the various features of the island syndrome may be expected to occur in a fairly regular chronology, with each change being the precursor to subsequent changes in characteristics of the insular lineage^7^. Once established, the descendants of a founding population may undergo rapid increases in their populations, quickly followed by expansions into habitats, diets, and other ecological strategies considered atypical for the species on the mainland (invading niches left vacant in the absence of their mammalian competitors and predators)^8^. Following these early phases of the island syndrome, which may occur within just a few generations, descendants of these initial populations may undergo phyletic evolution which allows them to progressively adapt to their new niches and insular environments^8, 10^.

For large mammals, with their relatively long generation times, the latter, evolutionary phases of the island syndrome may be expected to require extremely long periods of ecological isolation. There is, however, some limited but intriguing indication that at least the initial stages of dwarfism in very large mammals can be remarkably rapid (e.g., some artiodactyls and proboscideans decreasing to between 25–50% of the mass of the ancestors in just a few millennia)^5, 6^. Lister hypothesized that this represented the first of a two-stage process of body size evolution in large mammals – this first, very rapid stage being followed by one of slower but more prolonged dwarfism which, providing persistence of the insular lineage, often produced the true marvels of island evolution including hippos and elephants that dwarfed to just 5% of the mass of their mainland ancestors^10^. Lister’s initial stage of dwarfing is characterised by an interplay of plastic and genetic effects^10^. In particular, the genetic component of size reduction would come about in part by genetic assimilation of initial ecophenotypic stunting. This stage is dominated by strong selective pressure for size reduction and it is accompanied by allometric and other developmental effects^10^. In the second stage, mostly driven by natural selection, processes already begun in the initial stage continue much longer and produce more extreme changes^10^. A common feature in dwarfed island herbivores is a great shortening of the limbs, especially the distal limb elements. This has been explained as an adaptation for what Sondaar (1977) described as low-gear locomotion^9^ – a frequent phenomenon believed to be more adaptive, in the absence of predators, for climbing across rocky and hilly terrain than the ancestral (mediportal) bauplan.

While the great wealth of evidence on body size evolution in large mammals is derived from palaeontological studies, anthropogenic introductions of mammals to islands in recent history can also provide key insights into the rate and nature of the island rule and, more generally, the island syndrome. Such introductions can serve as valuable manipulative experiments on the ecology and evolution of isolated biotas. Here, we describe the case of the feral cattle (*Bos primigenius taurus*) of Amsterdam Island (South Indian Ocean), which provides us with a rare opportunity to assess the rapidity of demographic, life history, and evolutionary (body size and bauplan) responses of large mammals to the ecological simplicity of a very isolated, insular environment.

**Figure 1:**
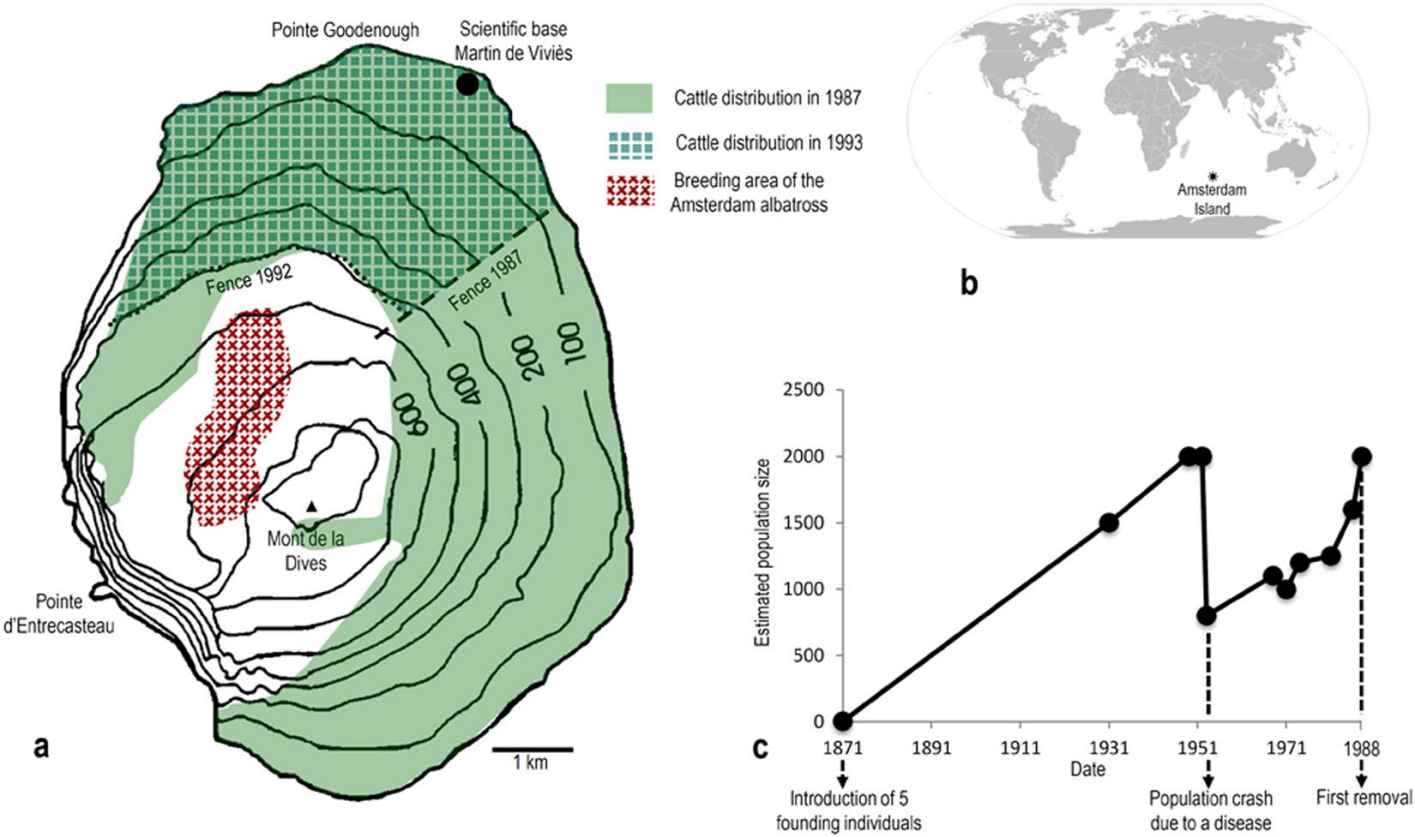
Map of Amsterdam Island and general information about the feral cattle population. (**a**) Feral cattle distribution and breeding area of the Amsterdam albatross (map modified after ref. 16). (**b)** Location map of Amsterdam Island (map modified from Wikimedia Commons, public domain: https://commons.wikimedia.org/wiki/Maps_of_the_world#/media/File:BlankMap-World6.svg). (**c)** Feral cattle population size from introduction to first removal (data from ref. 16).

Amsterdam Island (37°40′33″S, 77°33′17″E) is a 55 km^2^ volcanic dome in the South Indian Ocean that arose between 0.4 to 0.2 myBP^11^ (Fig. 1). At the time of introduction of the cattle, its maximum elevation was 881 m above sea level and the island habitat ranged from *Phylica nitida* trees and grasses in the lowlands to dwarf shrubs (*Acaena magellanica*), sphagum bogs and mosses along the higher elevations^12^.

The feral herd of *Bos primigenius taurus* was established when five individuals were introduced onto the island in 1871, these being translocated from Réunion Island (21°06′52″S, 55°31′57″E). These individuals were descendants of French stocks present on Réunion Island, which at that time included the following breeds: Jersey, Tarentaise, Grey Alpine, and Breton Black Pied^12–15^.

From the initial founders, this unmanaged population, free from artificial selection and natural predators and competitors, quickly increased in number (Fig. 1) and spread across the island to occupy most of the lowland habitats (those <500 m), which included grasslands, shrubs, and *Phylica nitida* trees and ferns. By 1950, the population had increased to an estimated 2000 individuals – sufficient numbers such that grazing and trampling pressures heavily impacted the island’s native biota. In fact, at that time much of the native vegetation had disappeared in favour of introduced plants and, particularly, forage grasses^16^. The population of feral cattle then experienced a collapse in 1951 due to an undetermined disease outbreak, but quickly rebounded to reach pre-collapse levels by the 1980s^16^. With the ecological impacts of the burgeoning population of cattle now threatening the survival of island endemics, including the endemic tree (*Phylica nitida*) and the Amsterdam Island albatross (*Diomedea amsterdamensis*), conservation measures were initiated to control the herd^16^. This included erection of fences in 1987 and 1992 to restrict the range of the cattle (Fig. 1), and culling, which began in the late 1980s but, ultimately, was followed by extermination of the herd in 2010 to protect native plants and animals.

## Results

**Figure 2:**
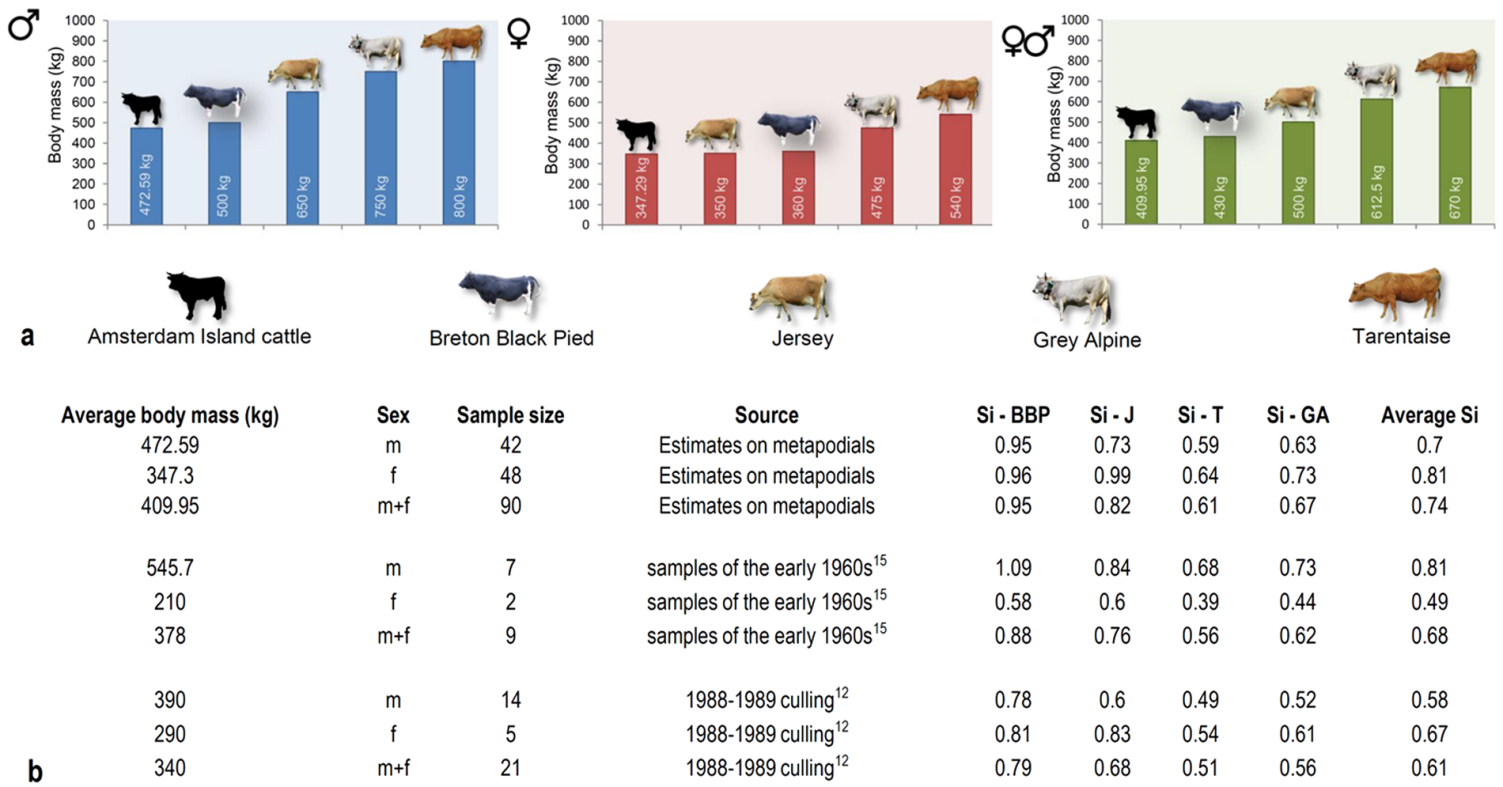
Body masses of adult Amsterdam Island cattle and body size divergence with respect to ancestral French breeds. (**a**) Comparison of average body masses of adult Amsterdam Island cattle to that of the ancestral French breeds. Silhouette of cattle from Amsterdam island drawn by R.R. Images of other breeds from Wikimedia Commons, user credits from left to right: https://en.wikipedia.org/wiki/Bretonne_Pie_Noir#/media/File:Taureau_breton_pie_noir.JPG by Gaëlle Diabaté is licensed under CC BY-SA 3.0 (https://creativecommons.org/licenses/by-sa/3.0); https://en.wikipedia.org/wiki/Jersey_cattle#/media/File:Walworth_Gate_010.jpg by Storye book is licensed under CC BY 3.0 (https://creativecommons.org/licenses/by/3.0); https://de.wikipedia.org/wiki/Tiroler_Grauvieh#/media/File:Tiroler_Grauvieh0005.jpg. by Hausegger is licensed under CC BY-SA 3.0 AT (https://creativecommons.org/licenses/by-sa/3.0/at/deed.en); https://de.wikipedia.org/wiki/Tarenteser_Rind#/media/File:Tarentaise2.jpg by Cyrille Bernizet is licensed under CC BY-SA 3.0 (https://creativecommons.org/licenses/by-sa/3.0). For details about body mass estimates see Methods. (**b**) Body size divergence (Si = mass of insular individuals / mass of mainland individuals) of feral cattle from Amsterdam Island with respect to ancestral French breeds (BBP: Breton Black Pied; J: Jersey; T: Tarentaise; GA: Grey Alpine). Different values are based on body mass estimates obtained from metapodial dimensions of 90 specimens culled in the first removal (1988–1989)^14^ and from direct measurements of individuals reported in the literature for samples taken in the early 1960s^15^ and from the 1988–1989 culling^12^. Average Si is calculated with respect to an estimate obtained averaging body size values of all the ancestral French breeds.

Our estimates of body size divergence^17^ (Fig. 2) are based on body mass estimates obtained from metapodial dimensions of 90 adult individuals culled in the first removal (1988–1989)^14^, using allometric regression models proven to be optimal for estimating body mass for Bovidae^18^ (see Methods). Our body mass estimates are only partly in agreement with direct measurements of individuals reported in the literature for samples taken in the early 1960s^15^ and from the 1988–1989 culling^12^ (Fig. 2). These differences most likely reflect the significantly larger dataset on which our estimates are based^14^ (Fig. 2). To express body size divergence as relative reduction from the mass of the founding, ancestral individuals we assumed that their size was representative of that for the ancestral breeds, and then calculated the average body mass for those breeds (Breton Black Pied: 430 kg; Jersey: 500 kg; Grey Alpine: 612.5 kg; Tarentaise: 670 kg; see Methods).

**Figure 3:**
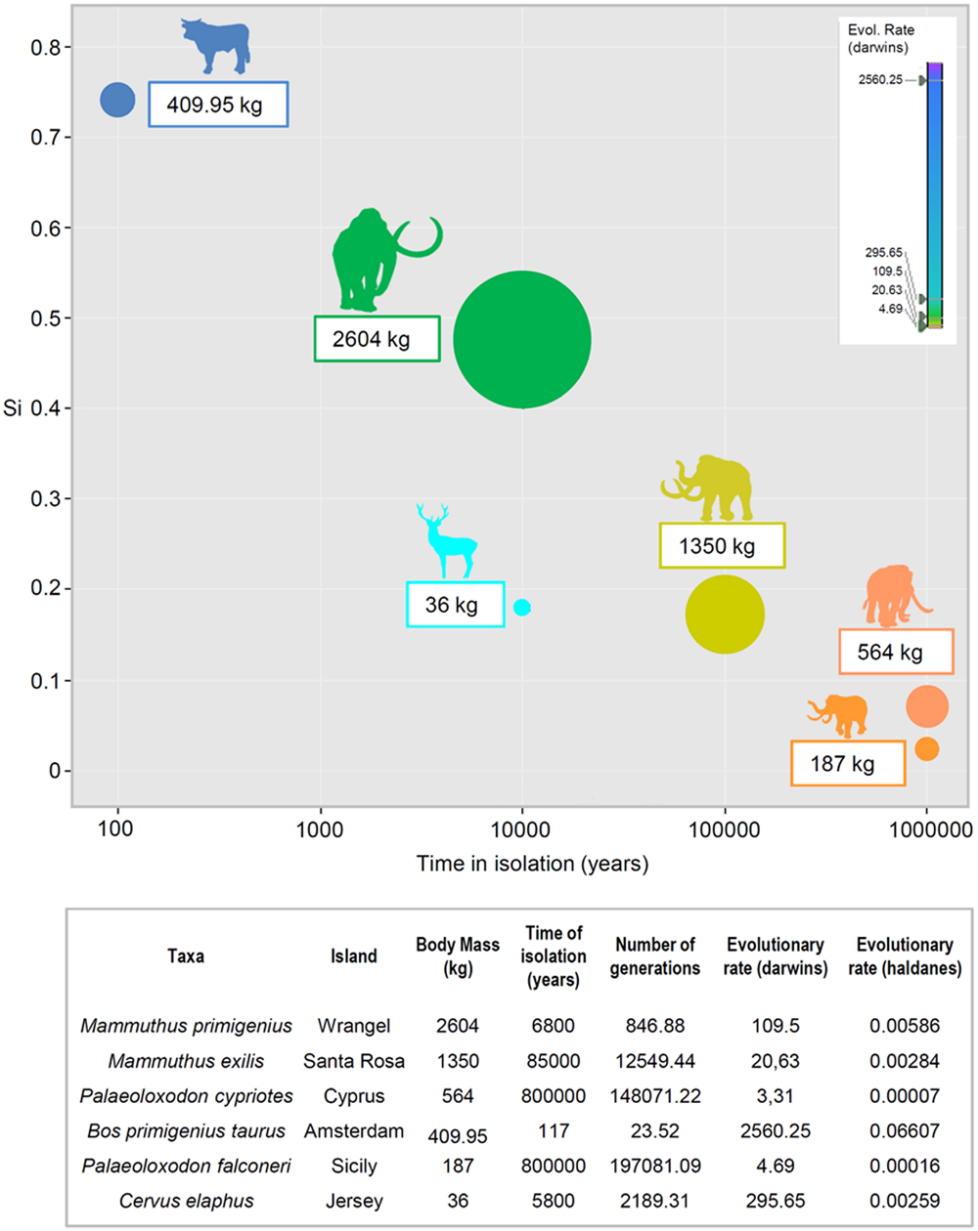
Evolutionary rates of body size reduction of Amsterdam Island cattle and other insular large mammals. Evolutionary rates of body size reduction of Amsterdam Island cattle expressed as functions of time in years of isolation (darwins)^19^ and number of generations (haldanes)^21^. Evolutionary rates of other extreme cases of body size reduction in insular large mammals are given for comparison and represented by bubbles of different colours in the chart (silhouettes of focal species drawn by R.R.). The size of each bubble is proportional to the body mass of each species/population. It is noteworthy that evolutionary rates of *Bos primigenius taurus* from Amsterdam Island are more than an order of magnitude higher than any other cases reported in the literature. See Methods and Discussion for details about calculation of evolutionary rates and for a more conservative estimate of rates based on founding of Réunion Island (which nonetheless yields extremely high rates of dwarfism, 927.4 darwins and 0.02393 haldanes).

As illustrated in Fig. 2, the Amsterdam cattle are smaller than the ancestral French breeds, including the small Breton Black Pied. Results of one-sample t-tests (see Supplementary Table S1) confirm a significant body size reduction of Amsterdam Island males and females with respect to average reference values obtained for the ancestral population (see Methods). Our estimates of body size reductions in adult Amsterdam Island cattle (using average Si values; see Methods) ranged from 49% to 81% of the mass of the ancestral stock, thus suggesting that, although substantial, their divergence was in the latter periods of Lister’s first stage of insular dwarfism of large mammals (i.e., in comparison to the extreme cases of reduction [often >80% mass reduction achieved during prolonged periods of Lister’s second stage])^10^. Thus, as Lister asserted, the initial stage of insular dwarfism can occur very rapidly indeed – in this case within just 117 years (about 24 generations according to our estimates; Fig. 3) from initial establishment to the culling and sampling period of 1988. The corresponding evolutionary rates (2560.25 darwins and 0.07 haldanes for Amsterdam Island cattle), based on our body mass estimates, are among the highest reported in the literature for dwarfing in mammals^19–22^ and, despite likely accounting for the contributions of other factors to the dwarfing of the focal population (see Discussion), attest to the ability of large mammals for rapid morphological adaptations to insular environments (Fig. 3). Alternative, and even higher, values of evolutionary rates were calculated on the basis of direct body weights of individuals reported in the literature^12, 15^ and they are included in Supplementary Table S2.

## Discussion

The most rapid and well documented cases of island dwarfing known thus far took place over thousands of years and include red deer from Jersey, Channel Islands^5^, and mammoths from Wrangel Island, in the Siberian Arctic^6^. Dwarfing of cattle from Amsterdam Island occurred in an exceptionally short time of isolation, since only 117 years passed between its introduction and the first removal in 1988.

Consistent with the general patterns of the island syndrome, the marked reduction in body size of Amsterdam Island cattle was accompanied by, and likely preceded and perhaps facilitated by demographic and ecological release. Population densities of these cattle rose to extremely high levels in comparison to continental populations of feral ungulates, and occupied atypical habitats including shrub and forest (Table 1). Accordingly, the diet of the feral cattle became more diverse, including introduced (e.g., *Poa annua*, *Holcus lanatus*) and native grasses (e.g., *Poa novarae*, *Spartina arundinacea*), ferns (e.g., *Elaphoglossum succaesifolium*, *Gleichenia polypodioides*), and the endemic tree *Phylica nitida* ^12–16^. Amsterdam cattle also exhibited marked shifts in life history traits, including a variety of those associated with so-called fast life (formerly “r-selected”) strategies^23–25^, such as earlier age at first reproduction, expanded season of breeding, and female-biased mortality in comparison to continental populations^13, 26–28^ (Table 1). Likely closely associated with the habitat shifts referred to above, shortening of metapodials in these cattle^14^ indicates a marked shift towards low-gear locomotion^9, 29^.

**Table 1:** Population data of cattle from Amsterdam Island and selected feral herds.

	Amsterdam Island		Chillingham Park		Doñana National Park	
Latitude	37°40′33″S		55°31′31,64″N		37°N	
Date	1988		1989		1989	
Estimated herd size (ind.)	2000		52		191	
Density per total surface (ind./ha)	0.64		0.39		0.03	
Density per surface of available pasture (ind./ha)	0.75–0.91		—		0.04	
Seasonality of breeding	January–March		All year		May–November	
Age at first reproduction (months)	24–36 (minimum: before 15)		36–66 (minimum: 24)		—	
Sex ratio	1:0.797 (m:f)		1:1.95 (m:f)		1:2.56 (m:f)	
Age structure	0–2 years (21.8%);	0–2 years (32.3%);	0–2 years (30%);	0–2 years (28.2%);	0–4 years (66.67%);	0–2 years (21%);
	2–5 years (17.9%);	2–5 years (31.3%);	2–5 years (20%);	2–5 years (10.3%);	>4 years (33.33%)	>3 years (79%)
	>5 years (60.3%)	>5 years (36.3%)	>5 years (50%)	>5 years (61.5%)		
Mortality of juveniles	22.40%	11.70%	50%		—	
Mortality of adults	18.30%	47.70%	—		—	

Population data of cattle from Amsterdam Island^12, 13^ in comparison to the herd of Chillingham (in northern England)^26^, frequently used as control sample in population studies of insular cattle^12, 52^, and the feral, continental herd from Doñana National Park (SW Spain)^53^. Data about age structure and mortality are given separately for males (left half-columns) and females (right half-columns), when available.

Although our estimates of body size reduction and evolutionary rates are based on an accurate database, some unavailable details introduce a minor degree of uncertainty. In particular, a few details about the origin and size of the founding individuals, and the time of their introduction on Amsterdam Island need to be discussed further.

For example, we assumed that the founders of Amsterdam Island population were not already dwarfed, though they derived from another insular population – that of Réunion Island. One of the reasons behind this assumption is that the initial livestock populations on Réunion Island were hunted out by the mid-nineteenth century^30^, about 20 years before the introduction of the cattle on Amsterdam Island. Therefore, the 5 founding individuals of the Amsterdam Island population would have been selected from stocks re-introduced on Réunion Island not earlier than the mid-nineteenth century. Accordingly, a hypothetical dwarfing of the Réunion Island population would had occurred in just a couple of decades, from about 1850 to 1871 (the date of introduction on Amsterdam Island). Furthermore, there is no mention in the literature of a possible dwarfing of cattle from Réunion Island since European occupation of the island in 1665.

The hypothesis that some individuals of zebu cattle, *Bos primigenius indicus*, may have also been introduced on Amsterdam Island, although mentioned by a few authors^14, 31^, was never confirmed. This hypothesis, based on putative similarities in the frequencies of haemoglobin variants shared by the Amsterdam Island cattle and African and Malagasy taurine and zebu breeds^32^, was dismissed by Lesel, who did not observe any morphological trait typical of zebu bovines in the individuals of the focal island population^15^. In light of the results of genetic studies, which highlighted similarities in the distributions of haemoglobin allele frequencies between a few taurine breeds (in particular, the Jersey breed) and zebu breeds^33^, and the diversity of haemoglobin variants and complexity of their geographical distribution among zebu and cattle breeds^34^, we agree with Lesel in ruling out the hypothesis of a zebu ancestry for the Amsterdam Island cattle.

Our average estimates of body size reduction and evolutionary rates for the focal cattle rest on the assumption that the founding population included individuals of all the ancestral French breeds present on Réunion Island at the end of the 19th century (see Methods). The highly diversified coat colours and patterns exhibited by the individuals of Amsterdam Island cattle^12, 14, 15^ are in agreement with this assumption. In particular, the high percentage (about 84%) of fawn- and sand-coloured individuals in the focal insular population^15^ would exclude a mostly Breton Black Pied ancestry, or, at least, confirms the occurrence of larger and fawn-coloured (Tarentaise), light-coloured (Grey Alpine, Jersey) breeds among the 5 founding individuals.

Our reference body size values for these ancestral breeds come from a FAO report from the 1960s^35^. We relied on this old source to prevent our dataset from being affected by the last 60 or so years of artificial selection - likely selectively breeding for higher beef yields - on French farms. However, we are aware of the potential risk that the exact body masses of the ancestral breeds at the time of colonisation of Amsterdam Island might have been slightly smaller than our reference values^35^, due to the consequences of any artificial selection to increase size of the focal French breeds in their home range throughout the first half of the 20th century. Nevertheless, available evidence suggests that this bias would not be significant. In particular, animal husbandry became very intensive only from the 1960s, and artificial selection pressure for large framed cattle was not common in the first half of the 20th century^36^. Conversely, at that time, cattle in Europe and the Americas (including the ancestral French breeds in this study) were utilised primarily for milk and draught and, until the late 1950s, artificial selection pressure for early maturing, small framed cattle was the major trend^36^.

The possibility of a previous introduction of cattle of European origin on Amsterdam Island by the first colonizers in the 17th century is cited in the literature^15^ (although only once and it is considered less probable by the author), but it has been dismissed^37^. Nevertheless, we produced a more parsimonious estimate of evolutionary rates, using an increased time period of 323 years (considering the founders of the Réunion Island population as the founders of the Amsterdam Island one and taking as starting point the year of colonization of Réunion Island). Even using this prolonged time period, we obtained rates of dwarfism that are significantly higher than the ones that we obtained for other large mammals (Supplementary Table S2 and Fig. 3). The body size reduction of Amsterdam Island cattle is an example of rapid dwarfing in an allopatric isolate and was probably the result of a combination of factors.

Artificial selection experiments continue to show the potential for fast rates of evolutionary change in different organisms^38, 39^. Cattle have the capacity for such rapid rates when subject to strong (albeit artificial) selection pressures^40^. It appears, however, that the population of Amsterdam cattle was not influenced by artificial selection^14^, and the only human interference was the removal of 50–80 individuals each year, mainly adult and subadult males, to provide fresh meat for people living on the island^12^. Furthermore, this hunting regime started only in recent times when the scientific base Martin de Viviès was built (1949). In order to isolate the effect of other factors and to avoid biased information, we used (in all possible cases) data coming from Zone I^12^, an area located in the southern part of the island, where cattle had never been hunted. Nevertheless, we cannot exclude the influence of artificial selection during immigration in triggering two potential, extreme bottlenecks during the founding of the Réunion Island and Amsterdam Island cattle populations. For instance, the hypothesis that 19th century sealers might have introduced mainly relatively small individuals, easier to transport and keep alive on wooden ships, on Réunion Island cannot be ruled out. Moreover, high livestock mortality during sea shipment, frequently exceeding 50% due to overcrowding, inadequate feed supply, and rough seas^41^, could have played an additional role in influencing the composition of the ancestral populations.

Island populations are usually characterised by lower average levels of genetic variation than mainland populations^42^. For instance, a founder effect and/or genetic drift have been invoked to explain the extremely low genetic diversity of feral cattle from Kuchinoshima Island, Japan^43^. This small population (<100 individuals) originated from a few individuals of Japanese native cattle imported on the island around the year 1900, and it exhibits unique genetic characteristics - including allelic distribution and peculiar frequencies of the genes associated with carcass weight - different from those of Japanese Black and Mishima cattle^44^ (the latter retaining the characteristics of native Japanese cattle). Significant inbreeding likely occurred also in the cattle population from Amsterdam Island and it might have driven its body size reduction. In particular, the disease outbreak and resulting population collapse experienced in 1951 by the focal population likely triggered a third, significant bottleneck, in addition to the ones associated with immigrant selection at the time of colonization of Réunion Island and Amsterdam Island. Unfortunately, no detailed information about the nature of the disease is available and it is, thus, impossible to speculate whether it affected some individuals more than others. In fact, individual hosts in a population are characterised by different resistance and tolerance levels to specific infectious pathogens^45^.

Although it is widely accepted that genetic drift plays a key role in influencing the evolution of small, isolated populations and in decreasing their genetic diversity, other forces need to be taken into account. For instance, despite a strong founder effect, a significant increase in heterozygosity over time was recorded in an insular mouflon (*Ovis aries*) population from Haute Island (Kerguelen archipelago, Sub-Antarctic Indian Ocean)^46^. A longitudinal genetic survey highlighted that the changes in genetic variation observed in this population could be explained by disruptive selection, likely lowering the impact of drift on the loss of genetic diversity^46^. Recent phenotypic changes in wild mammal populations can arise due to environmental effects acting directly on an individual’s phenotype (plasticity) and on the maternal phenotype (maternal effects)^47^. Ozgul and coauthors^48, 49^ highlighted the higher ecological relevance of plasticity with respect to microevolution, namely genetic change across generations^50^, in body mass changes of Soay sheep from St. Kilda archipelago (Scotland, UK) and yellow-bellied marmots from the Upper East River Valley (Colorado, USA). Phenotypic plasticity is widespread in nature and different levels of plasticity may influence genetic evolution in different ways^51^. Accordingly, we cannot exclude that environmental effects - i.e., poor nutrition arising from limited resource availability, high competition due to high density - may have played a role in the dwarfing of Amsterdam Island cattle. In particular, shortage of food related to overgrazing could have affected the focal cattle population during the last decades before its extermination^12^. On the other hand, the high population density of cattle (see Table 1) would seem to argue for a healthy population and overall high carrying capacity of the environment, that was sufficient to sustain a successful herd (starting with just 5 individuals and developing quite fast, overcoming a disease and expanding again; see Fig. 1). Moreover, Lomolino and coauthors^3^, investigating the causality of the island rule, showed that resource limitation (as inferred from island area) does not influence body size shifts in large insular mammals.

Taken together, the divergence in morphological and ecological traits exhibited by the very recent, albeit short-lived, population of Amsterdam Island cattle attests to the substantial variability in these traits among individuals and to the power of ecologically simplified, isolated islands to drive rapid transformations of their biotas. In the absence of detailed, quantitative genetic data on the Amsterdam Island cattle population, it is not possible to confirm the relative influences of microevolution and plasticity underlying the observed body size reduction. Nonetheless, based on available evidence and on the evolutionary history of a similar, albeit less isolated, population^43, 44^ it seems probable that a combination of factors, including two or three extreme bottlenecks and subsequent inbreeding, absence of predators and competitors, and range expansion into highly atypical habitats may have driven this case of rapid dwarfism in a large mammal. This suggests one plausible, albeit largely untested, explanation for the differences in evolutionary rates reported for Lister’s two stages of body size evolution. The first stage might be largely driven by genetic drift and selection of the variation that already exists among individuals in founding populations, whereas the second more pronounced but much slower stage would require accumulation of new mutations and recombinations of genes that must be co-adapted and build on incrementally modified blueprints (body size, bauplan, and demographic, life history and ecological strategies) of evolving lineages.

## Methods

We estimated the body mass of the cattle of Amsterdam Island by using the predictive equations with the lowest predictive errors among those based on the available dimensions of metapodials^14, 18^ (Supplementary Tables S3–S4).

To infer the size shift of the focal population in light of the island rule, we collected available body size data of the ancestral breeds introduced on Amsterdam Island^35^ and we compared them with our estimates (Fig. 2). The relative size of the feral cattle of Amsterdam Island was expressed as a proportion of the body masses of the ancestral French breeds (Si = body size divergence^17^; Fig. 2). To obtain reliable estimates, we carefully selected the most appropriate reference body size values for the ancestral breeds from an old, but reliable report^35^. We avoided referring to more recent sources of data because they often include body size values that are more strongly affected by recent artificial selection and less likely to approach the size of the original introduced individuals. Furthermore, when regional size differences within the same breed exist, we adopted a conservative approach and selected the minimum available body size value (e.g., 350 kg for Jersey cows)^35^. Average Si values were obtained by comparison with the average body mass of the French breeds, and thus by taking into account all combinations of the 5 founding individuals. The estimate rests on the assumption that the founding population included at least one individual of each breed (see Discussion). We used two-tailed one-sample t-tests to compare these average body mass values estimated for the ancestral population (males: 675 kg; females: 431.25 kg) with distributions of body mass estimates of Amsterdam Island males and females, and to assess whether the body size reduction of the focal island population was significant (Supplementary Table S1). Distributions of Amsterdam cattle body masses included mean, minimum, and maximum estimates, based on selected dimensions of metapodials^14^ (Supplementary Table S1). Statistical analyses were performed using XLSTAT (version 2017.4, Addinsoft).

We calculated evolutionary rates of body size reduction of Amsterdam Island cattle and of the most pronounced examples of dwarfing reported in the literature among insular large mammals^4–6^ (Fig. 3). As in the case of body size divergence, to obtain average evolutionary rates of body size reduction of the Amsterdam Island population we compared the average body mass value of the focal population estimated from metapodial dimensions (409.95 kg), with the average body mass of the French breeds (553.13 kg). Although we believe that our body mass estimates of Amsterdam Island cattle, based on a rather large dataset^14^, are more reliable than the direct body weights of individuals reported in the literature for samples taken in the early 1960s^15^ and from the 1988–1989 culling^12^, we also obtained alternative estimates of evolutionary rates on the basis of the latter data (see Supplementary Table S2).

Evolutionary rates were expressed in darwins^19, 20^, (d), as (Log x2-Log x1)/Dt, where a trait evolved from x1 to x2 over a time Dt in millions of years. Log is the natural logarithm, and the variable x is, in this case, body mass. Rates in darwins can easily be calculated from mean values published in the literature, but they are inversely related to the time interval over which they are calculated^20, 21^. However, this scaling relation may be influenced by a mathematical artifact due to the plot of a ratio (rate) against its denominator (Dt)^20^. To avoid this scaling problem we also expressed evolutionary rates in haldanes^21, 22^, (H), as D/I, where D = d/s_p_. The proportional difference between the sample means (d = ȳ_2_ − ȳ_1_) is, in this case, the difference between the insular body mass and the ancestral body mass; s_p_ is the pooled standard deviation of the samples; I is the time interval between the samples, I = t_2_ − t_1_, estimated in generations. All the measurements of the samples were logged (logs to the base *e*) because of the geometric normality of biological variation^21^. Body mass standard deviation (s_p_) was estimated from published data^4–6, 14^ as (ln(maximum) − ln(minimum))/4, based on an estimate that 95% of normally-distributed observations are within two standard deviations of the mean^22^. The number of generations or biological time (I) experienced by the population is equal to the chronological time experienced divided by generation time G (Fig. 3). We estimated the generation time from our body mass estimates and literature data using an allometric scaling function developed for placental mammals^22^.

### Data availability

The authors declare that the data supporting the findings of this study are available within the paper and its supplementary information files.

## Electronic supplementary material

Supplementary Information
