# Supplementary material for: Rapid Dwarfing of an Insular Mammal – The Feral Cattle of Amsterdam Island
Source: Sci Rep. 2017 Aug 18;7:8820. doi: 10.1038/s41598-017-08820-2 (PMC5562861; doi:10.1038/s41598-017-08820-2)
Supplement: Supplementary file 1 — Supplementary Information [file 41598_2017_8820_MOESM1_ESM.pdf]

Supplementary Information for

Rapid Dwarfing of an Insular Mammal – The Feral Cattle of Amsterdam Island

Roberto Rozzi[1,*], Mark V. Lomolino[2]

[1] Museum für Naturkunde, Leibniz-Institut für Evolutions- und Biodiversitätsforschung, 10115 Berlin, Germany
[2] State University of New York, College of Environmental Science and Forestry, Syracuse, NY 13210, USA
*Correspondence: roberto.rozzi@mfn-berlin.de

Supplementary Tables S1-S4

Supplementary Table S1. Distributions of body mass estimates (kg) of Amsterdam Island cattle and results of one-sample t-tests.

Distributions of body mass estimates of Amsterdam Island cattle		
**Mc2 mean**	479.12	358.75
**Mc2 min**	430.70	296.34
**Mc2 max**	530.79	446.49
**Mc3 mean**	538.23	392.81
**Mc3 min**	463.57	323.72
**Mc3 max**	620.25	485.28
**Mc4 mean**	435.52	320.37
**Mc4 min**	392.26	265.35
**Mc4 max**	481.42	376.90
**Mt2 mean**	471.77	341.44
**Mt2 min**	421.85	256.95
**Mt2 max**	525.42	409.32
**Mt4 mean**	445.27	324.29
**Mt4 min**	400.24	253.73
**Mt4 max**	493.38	384.62

Results of two-tailed one-sample t-tests		
**t**	12.81984	4.661911
**P-value**	< 0.00001	0.000367
**df**	14	14

Distributions of body mass estimates (kg) of Amsterdam Island cattle: males (in light blue), females (in light red). Mean, minimum, and maximum estimates, based on selected dimensions of left metapodials[14] (Supplementary Table S3), are reported and used to build distributions of values for comparison purposes. Results of one-sample t-tests highlight a significant body mass reduction of Amsterdam Island cattle (P-value < 0.05) with respect to average body mass values estimated for the ancestral population (males: 675 kg; females: 431.25 kg).

Supplementary Table S2. Comparison of evolutionary rates of body size reduction of Amsterdam Island cattle on the basis of different times of isolation and average body mass values.

Taxa	Island	Body Mass (kg)	Time of isolation (years)	Number of generations	Evolutionary rate (darwins)	Evolutionary rate (haldanes)
Bos primigenius taurus	Amsterdam	409.95	117	23.52	2560.25	0.06607
Bos primigenius taurus	Amsterdam	409.95	323	64.93	927.4	0.02393
Bos primigenius taurus	Amsterdam	378	117	24.02	3253.76	0.04658
Bos primigenius taurus	Amsterdam	378	323	66.31	1178.61	0.01687
Bos primigenius taurus	Amsterdam	340	117	24.69	4159.3	0.14101
Bos primigenius taurus	Amsterdam	340	323	68.16	1506.62	0.05108

Supplementary Table S3. Available dimensions of metapodials[14] (mm) used for estimating body mass of Amsterdam Island Cattle.

**Dimensions of metapodials[14]**								
	Average	SD		Average	SD		Average	SD
Mc2 left	60.9	2.4	Mc3 left	38.9	1.8	Mc4 left	62.7	2.7
Mc2 right	60.9	2.5	Mc3 right	38.9	1.9	Mc4 right	62.7	2.7
Mc2 left	54.6	2	Mc3 left	34.8	1.2	Mc4 left	55.1	1.9
Mc2 right	54.1	2	Mc3 right	34.9	1.2	Mc4 right	55.2	1.7
	Average	SD		Average	SD			
Mt2 left	50	2	Mt4 left	57.7	2.2			
Mt2 right	50.6	1.9	Mt4 right	57.8	2.4			
Mt2 left	45.3	1.5	Mt4 left	51.4	1.7			
Mt2 right	45.3	1.5	Mt4 right	51.5	1.6			
**Description of variables**								
Mc2[18] (= McBp[14]) = Transverse diameter measured at proximal end of the metacarpal								
Mc3[18] (= McDp[14]) = Antero-posterior diameter measured at proximal end of the metacarpal								
Mc4[18] (= McBd[14]) = Transverse diameter measured at distal end of the metacarpal								
Mt2[18] (= MtBp[14]) = Transverse diameter measured at proximal end of the metatarsal								
Mt4[18] (= MtBd[14]) = Transverse diameter measured at distal end of the metatarsal								

Selected dimensions of metapodials (mm) from[14] used for estimating body mass of Amsterdam Island Cattle. Average value and standard deviation (SD) of each measurement are given: males (in light blue), and females (in light red). A brief description of each variable is also provided.

Supplementary Table S4. Predictive equations and body mass estimates (kg) of Amsterdam Island cattle.

Predictive equations for body mass[18]	r^2	%SEE	%PE
log(body mass)= 2.6495log(Mc2) + 0.6016	0.9529	31	20
log(body mass)= 2.8291log(Mc3) + 1.062	0.9402	36	22
log(body mass)= 2.3765log(Mc4) + 0.7443	0.9203	43	25
log(body mass)= 2.922log(Mt2) + 0.6162	0.9405	36	36
log(body mass)= 2.7421log(Mt4) + 0.5614	0.9418	35	35

Body mass estimates					
Mc2	Mc3	Mc4	Mt2	Mt4	Average
479.11	538.3	435.52	463.69	446.33	472.59
354.43	394.41	321.06	341.44	325.16	347.3
416.77	466.36	378.29	402.57	385.74	409.946

Predictive equations for the estimation of body mass based on available dimensions (cm) of metapodials[14,18]. Body mass estimates (kg) of Amsterdam Island cattle: males (in light blue), females (in light red) and average of both (in light green).
